# Incentive effects of cash benefit among low-skilled young adults: Applying a regression discontinuity design

**DOI:** 10.1371/journal.pone.0241279

**Published:** 2020-11-02

**Authors:** Helle Bendix Kleif, Jacob Nielsen Arendt

**Affiliations:** 1 VIVE—The Danish Center for Social Science Research, Copenhagen, Denmark; 2 The Rockwool Foundation Research Unit, Copenhagen, Denmark; University of South Australia, AUSTRALIA

## Abstract

In 2014, the Danish Government implemented an active labour market reform directed at unemployed young adults under 30 years of age with low educational qualifications. The reform replaced the (unemployment) cash benefits with a lower education benefit for many of the unemployed aged under 30 and obliged the low-skilled in this group to enrol in a regular general or vocational (VET) education program. This paper exploits the sharp discontinuity that occurs at age 30 to estimate the joint effect of higher benefits and the cessation of educational obligations on the share receiving cash benefits and the share enrolled in education. We estimate the effects by applying a regression discontinuity design. We report results for the group of low educated young adults and for subgroups facing different economic incentives. The results establish that reaching age 30 creates an incentive to apply for cash benefits, and we find strong evidence that a significant increase in the share of cash benefit recipients relates to a corresponding reduction in the share of young adults enrolled in education. When including subgroups the size of the effect increases, and the results demonstrate that the effects are strongest among previous education benefit recipients. This indicates that the results are mainly driven mainly by individuals reverting to cash benefits.

## Introduction

Around 15 percent of young adults in member countries of the Organisation for Economic Co-operation and Development have not completed upper secondary education [1], exposing them to increased risk of unemployment and potential long-term disadvantages. Moreover, once unemployed, most active labour market programs show only modest employment effects for young people with low educational qualifications [2]. Therefore, how to prevent low-skilled and unemployed young people from experiencing long periods of unemployment or inactivity is an open question.

This study estimates the effect of the reforms to the Danish cash benefit program introduced in 2014. The reforms are directed at young adult cash benefit recipients without a qualifying education (qualifying education refers to completed vocational upper secondary level education or training (VET) or higher). The policy reforms oblige low-skilled unemployed youth to enrol in a general upper secondary or VET educational program if they are assessed by their jobcentre caseworker as being able to take part in a regular program, or to participate in individually tailored support that prepares them to enter education if they are not assessed as being education-ready. The transfer to education is incentivised economically by means of a lower cash benefit (the so-called education benefit), which is equivalent to the state study grants available to all students enrolled in at least upper secondary education [3]. The cash benefit program only applies to young adults below the age of 30, and we use this age cut-off point to estimate the effect of the reform using a regression discontinuity (RD) design. The unemployed aged 30 and over are not subject to the educational obligations and they are eligible for a means-tested monthly cash benefit that is up to 76 percent higher.

The primary focus of the study is on the potential relationship between the share enrolled in education and the share receiving cash benefits. A number of studies have examined the incentive effects of income transfer programs in general and for single mothers in particular [4–7]. However, we are only aware of five studies that have evaluated the effects on young unemployed persons [8–12]. As in the current study, the previous studies all apply an age-dependency in cash benefit eligibility to identify the effect of changes in cash benefit levels on different social outcomes. Four of the studies find that an increase in cash benefit levels leads to a significant but small reduction in employment rates or an increase in cash benefit dependence. While such a response is to be expected, it does not necessarily reflect the full response, since a large share of young persons may be already in, or change to alternative states, notably education. This is supported by the fifth study, which finds that the policy considered in the current study, saw an increase in the number of young adults who started an education when the cash benefit was reduced [12].

Therefore, we add to this sparse literature by examining the impact on enrolment in education of an employment policy that reduced cash benefit levels and introduced an education or training obligation for the education-ready under-30 age group. We also explore potential within group differences for subgroups of young adult cash benefit recipients subjected to different levels of economic incentives when turning 30. Like four of the previous studies, we use a strong causal research design [8–11], but in contrast to these, we supplement their parametric estimation approach by using a more flexible non-parametric RD estimator [13].

The study is organized as follows: In the next section we describe the reformed cash benefit program for young low-qualified adults. The following section describes the empirical strategy, and the fourth section describes the data. Section five contains the results and they are discussed and summarized in the final section six.

### The reformed cash benefit program

The Danish social assistance system is a government funded and means tested transfer program for the unemployed who do not have unemployment insurance. Eligibility for assistance, in the form of cash benefits, depends on whether the individual has other sources of income, assets or savings. The system has undergone extensive changes since the mid-1990s that have steadily increased the means testing and tightened eligibility criteria.

In 2014 the reformed cash benefit program came into force for unemployed young adults aged under 30 who had no formal qualifications. The aim of the program is to improve the skill set of this group by directing them towards education and training. To achieve this, the program applies two main measures: 1) The unemployment cash benefit was replaced with a reduced so-called “education benefit”, which brought the benefit payment into line with the state study grant, and 2) An educational obligation was introduced, requiring recipients to take part in certain types of education or training programs in order to be eligible for education benefits. Whereas the educational obligation applies to all benefit recipients below age 30, the reduced payment amount only applies to recipients who are assessed as being *education*-*ready*, i.e., it does not apply to the *activity-ready* group, which consists of all those who are assessed as having severe problems besides unemployment. The assessment of education readiness is conducted by a caseworker at the local jobcentre when the young adult applies for benefits. We also include a group that does not receive cash benefits, and naturally, these individuals are not assessed by a jobcentre caseworker or affected by the educational obligations. This group is labelled *not-assessed*. We describe the policy implications and expected effects for these three groups in detail in the following.

### The education-ready

The education-ready are young adults who are assessed as being able to enrol in a regular program of education within one year. If they are considered to be able to enrol within a few months, they are labelled immediately education-ready. As the policy consequences of the reformed cash benefit program are the same for all the education ready individuals, we consider them jointly as one group.

Under the 2014 reforms, to be eligible for education benefits, the education-ready are obliged to enrol in and attend a regular education program as soon as possible. Furthermore, the education benefit payments are between 42.3 and 76.4 percent *lower* than the cash benefit payments for those aged 30 years or over. Table 1 shows the benefit levels for different groups of unemployed education-ready young adults before and after turning 30.

**Table 1:** Benefit levels for the education-ready in 2014, by living arrangements, parenthood and age.

(Education) benefit levels for the education-ready below age 30 (in DKK)		(Cash) benefit levels for the education-ready aged 30 and over (in DKK)		*Absolute difference below and above age 30 (in DKK)*	*Relative difference below and above age 30 (in percent)*
Living with parents	2,524	Not providing for children	10,689	*8*,*165*	*76*,*4*
Not living with parents, not providing for children	5,827	Not providing for children	10,689	*4*,*862*	*45*,*5*
Couple with children and cohabiting with another benefit recipient	8,196	Providing for children	14,203	*6*,*007*	*42*,*3*
Couple with children and cohabiting with others	5,857	Providing for children	14,203	*8*,*346*	*58*,*8*

The table presents the monthly benefit levels for education-ready young adults by living arrangements, parenthood and age. The table presents the absolute and relative differences in benefit levels prior to and after turning 30. Source: Own calculations based on The Danish Ministry of Employment [14].

The reduced education benefit levels prior to age 30 potentially create an economic incentive among education-ready young adults to (re)enter the benefit system at age 30.

However, it is important to note that interventions introduced alongside the reduced benefits might affect the path of the outcomes prior to age 30. Since there are no education obligations after an individual has turned 30, it is difficult to distinguish between the effects due to economic incentives and the effects due to incentives induced by the educational obligations. Nevertheless, we expect to see behavioural effects when individuals within this group turn 30: All else equal they are inclined to drop out of education or employment and, to a greater extent, become dependent on cash benefits.

### The activity-ready

The category of activity-ready includes young adults with comprehensive problems besides unemployment, and therefore, they are not considered able to enrol in a regular educational program within one year [15]. Activity-ready young adults still have educational obligations that require them to enrol in a regular course of education or training (such as counselling and bridge-building courses to improve literacy, numeracy and work experience) organised by the local municipalities. For this group, active participation, or availability, releases a so-called “activity allowance” that offsets any differences in benefits prior to and above age 30 [3]. The only circumstance which influences the benefit level among this group is parenthood. However, as shown in Table 2, this does not affect the levels prior to or at age 30.

**Table 2:** Benefit levels for the activity-ready in 2014, by parenthood and age.

(Education) benefit levels for the activity-ready below age 30 (in DKK)		(Cash) benefit levels for the activity-ready aged 30 and over (in DKK)		*Absolute difference in levels when below and above age 30*	*Relative difference in levels when below and above age 30*
Not providing for children	10,689	Not providing for children	10,689	*No difference*	*No difference*
Providing for children	14,203	Providing for children	14,203	*No difference*	*No difference*

The table presents the monthly benefit levels for activity-ready young adult by parenthood and age. The table shows the absolute and relative differences in benefit levels prior to and after turning 30. Source: Own calculations based on The Danish Ministry of Employment [14].

Hence, there is no economic incentive at age 30 for these individuals, provided that they participated in, or were available for, active labour market interventions preparing them for education prior to turning 30. Accordingly, we do not expect to see behavioural effects stemming from economic incentives among activity-ready unemployed young adults when they turn 30. As with the education-ready group, there might be an effect from the educational obligation. However, we do not expect this to have a substantial effect on the share of the activity-ready group participating in education after they turn 30, because this would require them to have enrolled in education in the first place, which they are not obliged to do to nearly the same extent as the education-ready.

### Not-assessed category

Individuals in this category include young adults with low education who do not receive education benefits. They are included to test the extent to which the reforms to the cash benefit program affect individuals outside of the social assistance system, i.e., it provides indirect evidence on the inflow to cash benefit dependence at age 30, for a group who are not directly affected by the educational obligations.

## Empirical strategy

We apply the regression discontinuity (RD) design to estimate the causal effect of the reformed cash benefit program on outcomes of cash benefit receipts and educational enrolment when the individuals turn 30. The RD approach can be expressed formally in the following equation:

$$Y_{ia}=\beta_{0}+\beta_{1}{TREAT}_{ia}+\delta (a_{i})+\varepsilon_{ita}$$ (1)

Where *Y*_*ia*_ is the outcome for individual *i* of age *a*, where *a* is measured in number of weeks from the week when turning 30. As we use longitudinal weekly data, each individual is observed at values of *a* ranging from -100 to 100. *δ*(*a*) captures the relationship between age and the outcome variable in the absence of treatment, while *TREAT*_*ia*_ is a dummy variable that takes the value of 1 for individuals aged 30 or above (i.e., when *a* ≥0) and the value of 0 for individuals aged below 30, and therefore, *β*_1_ is the treatment effect of interest.

The key assumption in the RD design is that *δ*(*a*) is continuous around the threshold, i.e., that the relationship between age and outcomes, such as cash benefit receipt and educational enrolment, develops continuously with age in the absence of a reformed cash benefit program. This assumption isolates the only source of discontinuity around the threshold to be the treatment, and allows us to interpret *β*_1_ as a causal effect of the reformed cash benefit program [11]. Another way to express this assumption is that individuals who just turned 30 share the same set of characteristics and have similar outcomes as individuals just below age 30, who received the treatment. We have strong empirical evidence that cash benefits, employment and education depend on age, but see no reason why this should be discontinuous at age 30 in the absence of treatment. The estimated effect of turning 30 might, however, be affected by anticipation effects and delayed response time. This would show up as a response to the policy change prior to or after reaching age 30. Both would, however, bias the coefficients towards zero and thus *underestimate* the full impact of the reformed cash benefit program. To examine such a scenario, we allow for delayed response in some of the analyses below by excluding a short period of 20 weeks just after turning 30.

Since we do not know a priori how outcomes develop with age, we present estimates of the treatment effect using four different specifications of the regression function. They include first, second and third degree polynomial functions on each side of the threshold, as well as local linear regressions using the rdrobust function in Stata [13]. For instance, when using a first order polynomial, *δ*(*a*_*i*_) reads:

$$\delta (a_{i})=\delta_{1}a_{i}+\delta_{2}a_{i}*{TREAT}_{ia}$$ (2)

Therefore, all specifications allow outcomes to develop differently with age before and after turning 30. The local linear regressions determine how wide a window of data around the threshold that is used in a data-driven way, which allows for a much more flexible age dependence than the parametric polynomials, but comes at a cost of larger standard errors.

Finally, another reason for potential biases is that other policies affect the estimates at the age threshold. Therefore, we stress that, apart from the reformed cash benefit program, the period under study involves no other relevant changes in labour market policies, and no other labour market policy utilises age 30 as a cut-off. Regarding education policies, the Danish Government implemented a VET reform in August 2015 [16]. Among other measures, the reform increased the entrance requirements for admission to vocational education. From the perspective of the reformed cash benefit program, this might counteract the intention and efforts to bring low-skilled benefit recipients (back) into education prior to age 30. We do not consider that this policy change introduces biased estimates at the age threshold. But the fact that increased entrance requirements were introduced at the same time as low-skilled benefit recipients were pushed towards education, emphasises the relevance of interpreting any discontinuities at age 30 as the joint effect of the cessation of educational obligations and higher benefit payments.

## Data, population and descriptive statistics

We base the empirical analyses upon the DREAM register provided and administered by the Danish Agency for Labour Market and Recruitment [17]. This register contains weekly information on all public transfer payments, for all individuals who receive transfer payments, such as cash benefit, education benefit, state study grant, sickness benefit, maternity benefit and retirement benefit. The register enables us to follow each individual on a weekly basis in a longitudinal manner both before and after the age of 30.

We use weekly information to determine outcomes of interest. To establish the proportion who are enrolled in education at any time, we use weekly information on the number of individuals receiving state study grants. All students enrolled in tertiary education, and all those who are aged 18 and over and enrolled in upper secondary education, are eligible for study grants. The study grants were 5,903 DKK in 2015 [18], i.e., they were the same size as the education benefits for unemployed singles without children below 30 who are assessed as being education-ready (cf. Table 1). The share of cash benefit recipients is measured using information on individuals receiving cash benefits (when age 30 and above). For weeks where individuals do not receive any transfer payments, it is possible to differentiate between them being in employment (registered at a monthly level) or not, where employment is defined by having positive labour income. We refer to persons with no transfer income or employment as being self-supported (i.e., they are supported by means other than employment or transfer income). The DREAM register also contains information on the assessment categories into which the individuals are allocated by the caseworker. This is used to distinguish between the three groups with different treatments and incentives described above (the education-ready, the activity-ready and the not-assessed category). We use the individuals’ assessed category from the first quarter in which they receive education benefits, if they receive education benefits before they turn 30. Those who do not receive education benefits before they turn 30 are categorized as not-assessed.

To qualify the interpretation of the relationship between the share enrolled in education and the share receiving cash benefits, we combine the DREAM register with longitudinal registers on educational activity, which are administered and provided by Statistics Denmark. Those registers enable us to determine the reason why an educational activity ended, whether this was due to completion or dropout and, in addition, which type of educational activity the individual exits from. Finally, we merge this dataset with registers, also maintained by Statistics Denmark, that contain information on socio-demographic characteristics and family status. All data used are collected for administrative purposes and, therefore, benefit from not being subject to recall bias, attrition (with the exception of death and migration) and they have limited missing data.

### Population

We focus on young adults who turn 30 years of age during 2014 and 2015 and observe them in 2012 to 2016. This enables us to follow all young adults in the sample for at least 52 weeks before and after age 30. We have access to a randomly drawn 10% sample of this population, consisting of 13,413 young adults.

Our analysis sample consists of the sub-group of 4,936 individuals who are in the target group of the reformed cash benefit program: Young adults without a qualifying education (VET or higher). Each of the 4.936 individuals “at risk” of being affected by the reformed cash benefit program, is followed weekly prior to and after turning 30. The total longitudinal dataset contains 918,332 observations. Within the group of young adults at risk, we distinguish between the education-ready (n = 403), the activity-ready (n = 474) and those in the not-assessed category (n = 4,059). Before we present the estimated effects of the policy, we present the mean outcomes before and after turning 30. These are not meant for causal interpretations, but provide a broader view on the trend of the outcomes of cash benefit receipt and educational enrolment.

### Cash benefit dependence

**Fig 1:**
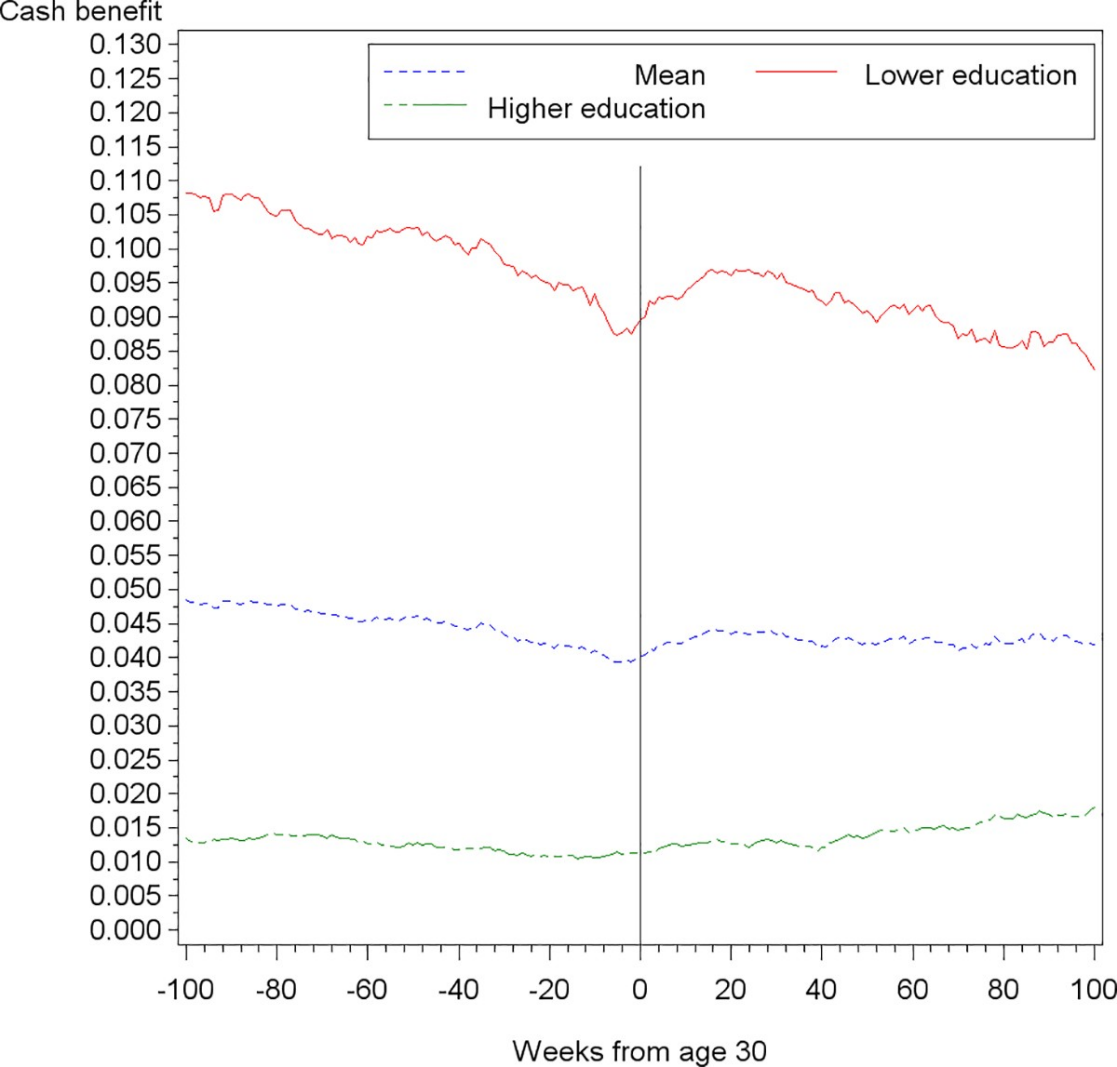
Mean share of cash benefit recipients by education level.

To give a first impression of the trend in the share of cash benefit recipients, Fig 1 compares the share within the main sample of low-skilled young adults with the share among young adults holding at least vocational education or higher. Each individual’s education level is observed in the week before turning 30. Week 0 indicates the week within which they turn 30. For simplicity we refer to mean shares of cash benefit recipients although individuals formally receive “education benefits” when they are below age 30.

As is evident from Fig 1, the percentage of cash benefit recipients among young adults with lower levels of education far exceeds the percentage among those with higher educational qualifications. In the week before turning 30, 8.9 percent of the lower educated group received cash benefits, whereas the average in the group with higher education was around 1.1 percent. Looking more closely at the trend of among young adults with lower levels of education (n = 4,936), the red line in Fig 1 depicts a rather steady decrease in the share of cash benefits recipients throughout almost all of the period leading up to week 0. With the dataset following the same individuals before and after age 30, the period prior to week 0 includes the young adults at ages 28 and 29, a period in which those receiving cash benefits (in the form of education benefits) will have been affected by the reduced benefit introduced at the implementation of the reformed cash benefit program on 1 January 2014. As Fig 1 illustrates, the decrease clearly ends at age 30, where it is followed by an increase in the share of cash benefit recipients. This trend provides a first indication of the consequence of the status change of individuals when they turn 30, i.e., the associated entitlement to a higher benefit payment and the simultaneous end of educational obligations. The absence of a similar trend among individuals with higher educational qualifications, who are not subject to treatment, supports this interpretation, as evidenced by the green line in Fig 1.

**Fig 2:**
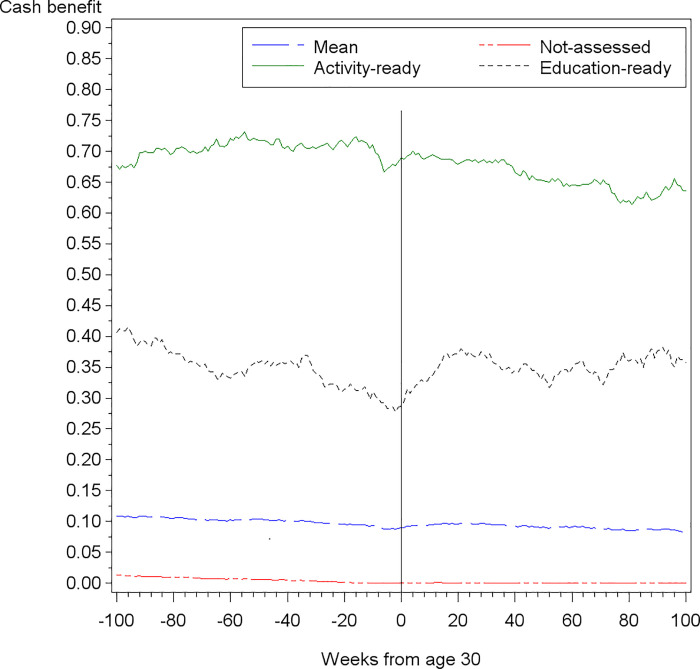
Mean share of cash benefit recipients among young adults with low qualifications, by assessment category.

Including data from the DREAM register on assessment categories, Fig 2 illustrates the share of cash benefit recipients before and after turning 30 for young adults categorized as education-ready (n = 403), young adults categorized as activity-ready (n = 474) and the not-assessed group (n = 4,059). The figure indicates that the education-ready are the main drivers of the trend, which is plausible if the economic (or joint) incentives drive the change *and* the activity-ready participate in active measures.

Both Figs 1 and 2 suggest that the response at age 30 does not happen instantaneously. The increase in cash benefit recipients, beginning at age 30, does not peak until 16 weeks after turning 30 (see S4 Table). In the following period (between weeks 16 to 31) the increasing trend levels out, and after week 31 the share of cash benefit recipients starts decreasing. This indicates the importance of allowing for response time in the regression models. In the results section we report the estimates from allowing for 20 weeks of response time. In S5 Table we show how the estimates are robust to alternative response times in the interval, where the trend levels out, from week 16 to week 31. Finally, the difference in responses by assessment category also support our decision to estimate effects separately for subgroups.

### Education enrolment rate

**Fig 3:**
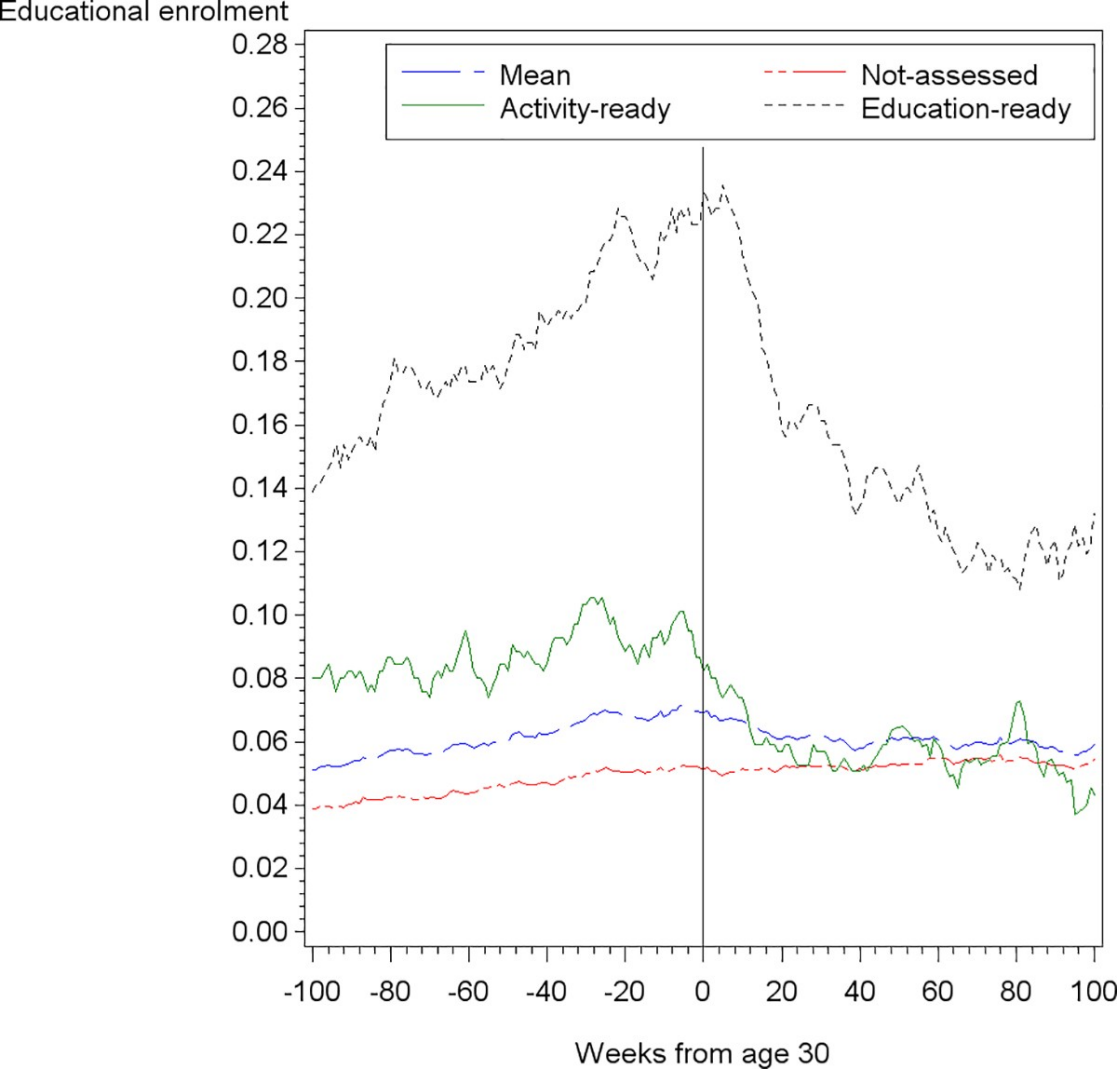
Mean education enrolment for young adults with low qualifications, by assessment category.

With the strong push towards education introduced by the reformed cash benefit program, education becomes a primary outcome. The dataset includes separate weekly information on education enrolment rate measured as individuals in receipt of state study grants. If the increase in the mean share of cash benefit recipients around age 30 shown above is related to educational enrolment rate, we would expect to see a decrease in rates of educational enrolment after reaching age 30. As Fig 3 depicts, we identify a clear change in education enrolment trends around age 30.

Fig 3 illustrates the trend in mean education enrolment among subgroups by the different assessment categories. Compared to young adults in the not-assessed category, we see a steep decreasing trend in educational activities shortly after turning 30, following a clear increase in the period just before. Regarding the activity-ready, we also witness a decline in educational activity close to turning 30. This trend is less steep but similar in timing to that of the education-ready, as expected. Similar to the development in the share of cash benefit recipients we witness a delayed response time with the decreasing education enrolment not taking off until about 5 months (20 weeks) after reaching the age-threshold.

Apart from education, other activities such as employment and being self-supported by other means might be associated with the trend in the share of cash benefit recipients, as illustrated in Fig 2. We explore this in S2 Table. It shows that the employment rate and level of self-support are also affected significantly by turning 30, however, to a lesser extent than the cash benefit receipt and education enrolment rates.

## Regression discontinuity estimates

In this section, we present the estimated effect of turning 30 on the shares enrolled in education and the share of cash benefit recipients. Our primary interest is to examine whether the path of cash benefit recipients mirrors the path of education enrolment rates. Table 3 reports the results of the RD estimates among young unemployed adults with low educational qualifications. We present all estimates with and without a delayed response time of 20 weeks and for different model specifications. In S5 Table, we demonstrate that the results are robust when using alternative response time. We report the results using a window width of 100 weeks (ages 28–31) on both sides of the age-threshold to fit the models. In S3 Table, we demonstrate that the results are also robust when using a window width of 50 weeks.

**Table 3:** RD estimates for young adults with low educational qualifications.

		First degree polynomial	Second degree polynomial	Third degree polynomial	Local polynomial (using Rdrobust)
**No response time**	Cash benefit	0.005*** (0.001)	0.006** (0.002)	0.005* (0.002)	0.004* (0.002)
	Education	-0.007*** (0.001)	-0.004** (0.001)	-0.000 (0.002)	-0.001 (0.002)
**Response time (20 weeks)**	Cash benefit	0.005*** (0.001)	0.008*** (0.002)	0.011*** (0.002)	0.009*** (0.002)
	Education	-0.010*** (0.001)	-0.011*** (0.001)	-0.009*** (0.002)	-0.007** (0.002)

The table presents the estimated effects of turning 30 on the share of cash benefit recipients and of those enrolled in education, i.e., *β*_1_ in Eq (1). Estimates are reported for the exact week where the individual turns 30 (= no response time) and when allowing for 20 weeks of adaption to the changes in payments and obligations (= response time of 20 weeks). The table presents the estimated effects using four different model specifications. Standard errors are given in parentheses

*** p<0.001

** p<0.01

* p<0.05. The included number of individuals = 4,936.

First, for the total population of young unemployed adults at risk, all specifications demonstrate significant effects of turning 30 on the share of cash benefit recipients—both when estimating the effect without and with response time. As expected, allowing for response time, and thus time to adapt to the reformed cash benefit program, increases the size of the effect. Without a response time, the immediate effect of turning 30 is to raise the share of cash benefit recipients significantly by around half a percentage point. When including 20 weeks of response time, the estimated effect is larger and equivalent to an increase of up to 1 percentage point.

Turning to the education enrolment, Table 3 shows that the model specification that does not allow for response time affects both the size of the estimated effects and their significance. This makes sense when considering the delayed decrease in mean education enrolment rates illustrated in Fig 3, because it is likely to take longer to register a drop out from education than from cash benefit receipt. The first and second-degree polynomial specifications without response time estimate an immediate decline in education enrolment rates of 0.4 to 0.7 percentage points. When response time is introduced, the results show a larger decline in education enrolment rates of up to 1 percentage point, thus corresponding to a simultaneous increase in the share of cash benefit recipients.

To qualify the interpretation of the effect on the education enrolment rate, we include information on the reasons for ending the educational activities after turning 30. We only include information on educational activities initiated after 1 January 2014, i.e., after the reforms to the cash benefit program were introduced. We present this information in Table 4 for each assessment category.

**Table 4:** Reason for ending educational activities among young adults with low qualifications by assessment category, (%).

Reason for exit:	Education-ready	Activity-ready	Not-assessed
Drop-out	68.6	74.2	36.8
Completion	31.4	25.8	63.2
N	51	31	68

The table presents information on the reasons for educational exits at age 30, for education activities initiated before turning 30 in the sampling period of 2014–2016. Source: Own calculations based on educational registers as well as the DREAM register.

Table 4 demonstrates how exits from educational activities due to drop-out seem to explain most of the decline in education enrolment rates among both the education-ready and the activity-ready. Of the activities that end at age 30, 69 percent of the exits among the education-ready and 74 percent of the exits among the activity-ready are due to individuals dropping out. In contrast, the drop-out rate is only 37 percent for those enrolled in education and who did not receive education benefits before turning 30. The education registers further show that the main activity not completed is vocational training (77–78 percent, not shown). Again, the increased drop-out rate might express the joint effect of higher benefits at age 30 and the simultaneously end of educational obligations. As for those who exit because of completion, the main educational activity is similarly vocational training (81–88 percent).

### Estimates by assessment categories

To establish further which young adults drive the above effects, we now consider effects for the three groups who are affected differently by the reformed cash benefit program: the education-ready, the activity-ready and those in the not-assessed category. Table 5 reports effects with and without response time.

**Table 5:** RD estimates for young adults with low educational qualifications, by assessment category.

		First degree polynomial	Second degree polynomial	Third degree polynomial	Local polynomial (using rdrobust)
***Not-assessed* (n = 4,059)**					
**No response time**	Cash benefit	0.001*** (0.000)	0.001** (0.000)	0.001** (0.001)	0.000* (0.000)
	Education	-0.002* (0.001)	-0.003* (0.001)	-0.001 (0.002)	-0.002 (0.002)
**Response time**	Cash benefit	0.001*** (0.000)	0.001** (0.000)	0.001* (0.001)	0.000 (0.000)
	Education	-0.001 (0.001)	-0.002 (0.001)	-0.000 (0.002)	-0.000 (0.002)
***Education-ready* (n = 403)**					
**No response time**	Cash benefit	0.045*** (0.007)	0.045*** (0.010)	0.040** (0.014)	0.017 (0.014)
	Education	-0.024*** (0.005)	0.003 (0.008)	0.011 (0.011)	0.017 (0.013)
**Response time**	Cash benefit	0.058*** (0.007)	0.074*** (0.010)	0.121*** (0.014)	0.082*** (0.014)
	Education	-0.072*** (0.005)	-0.060*** (0.008)	-0.065*** (0.011)	-0.048*** (0.011)
***Activity-ready* (n = 474)**					
**No response time**	Cash benefit	-0.007 (0.006)	0.018* (0.009)	0.006 (0.012)	0.025 (0.014)
	Education	-0.030*** (0.003)	-0.023*** (0.005)	-0.004 (0.007)	-0.010 (0.008)
**Response time**	Cash benefit	-0.020** (0.006)	0.006 (0.009)	0.015 (0.012)	0.016 (0.014)
	Education	-0.038*** (0.004)	-0.043*** (0.005)	-0.037*** (0.007)	-0.032*** (0.008)

The table presents estimated effects of turning 30 on the share of cash benefit recipients and educational enrolment rates among young adults by assessment categories. The estimates are reported for the exact week where the individual turns 30 (= no response time) and when allowing for 20 weeks of adaption to the changes in payments and obligations (= response time of 20 weeks). The table presents results using four different model specifications. Standard errors in parentheses

*** p<0.001

** p<0.01

* p<0.05.

First, Table 5 confirms that young adults in the education-ready and activity-ready categories, the majority of whom were affected by the policy change prior to age 30, almost exclusively drive the global effect on cash benefit receipt. Among young adults in the not-assessed category, the estimated effect on the share of cash benefit recipients is thus very close to zero across all model specifications. This result is robust with and without response time.

Next, for the group considered to be education-ready, the estimations across the first three specifications show a significant impact on the share of cash benefit recipients at the age-threshold of between 4.0 and 4.5 percentage points, whereas the non-parametric estimate is smaller and insignificant. The latter might be explained by the fact that the effect on the education enrolment rate does not show up in week zero. We confirm this when allowing for response time, where the increase in the share of cash benefit recipients increases to between 5.8 and 12.1 percentage points. The increase seems to be only partly explained by a corresponding decrease in the education enrolment rate of between 4.8 and 7.2 percentage points. S2 Table shows that other outcomes might explain the remaining effect: An estimated 1.9 to 3.5 percent leave employment, while 1.6 to 3.3 percent leave the self-supported status at age 30.

Among young unemployed adults categorized as activity-ready who participate in activation measures (see S1 Table), we do not expect to see an effect on outcomes for cash benefit receipt or educational enrolment at age 30. The estimates confirm our expectation, showing generally insignificant effects on the share of cash benefit recipients. This conclusion does not change when response time is included. There is, however, a significant negative effect on the education enrolment rate.

## Discussion and conclusion

We estimate the effect of a Danish active labour market reform that introduced a strong focus on active participation in education and training and reduced the amount paid in benefits to most unemployed individuals under the age of 30 who had no formal qualifications. The reformed cash benefit program brought the benefit payment levels for these individuals into line with the state study grant. The main aim was to increase the skill set of this group by creating incentives to take up or re-enter education or training. We identify the effect of the reform using the fact that it only applied to individuals below 30 years of age, and we contribute any discontinuous change in outcomes when turning 30 to the reform.

The study contributes to the sparse literature on the impact of income transfer programs on young unemployed persons. We demonstrate how the reformed cash benefit program affects young unemployed adults when they are very close to age 30. We find that the share of cash benefit recipients increases by 0.5 percentage points at the age-threshold for the total population of young unemployed adults with no qualifications. Allowing individuals time to adapt to the reformed cash benefit program further increase this effect by approximately 1 percentage point after 5 months. This small total effect corresponds to the size of the effect at age 25 found in a previous Danish study and a French study [9, 10], but is smaller than the effects found in an earlier French and a Canadian study [8, 11].

While previous studies focussed on the association between the economic incentives and either cash benefits or employment, we contribute to the literature by including enrolment in education as an outcome. This is important in any labour market policy context that includes a strong push towards education. Moreover, we distinguish the effects for groups with different assessment categories who are affected differently by the reformed cash benefit program.

Our contribution shows two main results: 1) Our results based on assessment categories demonstrate that the small total effect, also found in previous studies, masks much larger responses after the threshold point for groups of individuals who were already in receipt of (education) benefits, and 2) We demonstrate that the increased share of cash benefit recipients, to a large degree, is explained by a decreasing rate of enrolments in educational activities.

More specifically, with respect to 1): We show that the effect on cash benefit receipt is almost exclusively driven by young benefit recipients who were categorized as education-ready. These young adults were subject to both the reduced benefit payment amount and the educational obligation. In contrast, the effects are smaller for young benefit recipients who were not assessed as ready for education (activity-ready). This group was only subject to educational obligations, while the amount of their payments remained unchanged as long as they met their activation obligations. Finally, the effects are close to zero for low-skilled young adults who did not receive benefits before turning 30 (the not-assessed category).

These results speak strongly in favour of our hypothesis that the joint incentive from higher benefits and cessation of educational obligations affects the activities for young unemployed low-skilled adults. The results for the activity-ready show that the educational obligations have an impact on their own, but the larger effect for the education-ready shows that the impact is magnified when combined with changes in benefit levels.

With respect to 2): We find that the reformed cash benefit program increases the proportion of cash benefit recipients among the education-ready by 6 to 12 percentage points when allowing for response time—i.e., we find significant and much larger effects than have been previously found in total populations. We show that this increase corresponds to the decline in the share enrolled in education, those who are self-supported, and those who are employed, where the decline in the education enrolment rate is between 5 and 7 percentage points, i.e., more than half the increase in cash benefit receipt rates. The latter effect is similar in size to the effect reported in a previous study [12], which found that 6 percent more young adults enrolled in education at an earlier age due to the reformed cash benefit program.

The reforms to the Danish cash benefit program, introduced in 2014, reflect a political aim of incentivising unemployed low-skilled youth to take up education or training. When considering this aim in the context of our two main results, it highlights a need to reflect upon what defines meaningful educational obligations for unemployed youth close to age 30.

## Supporting information

S1 Table RD estimates on passive and active activity-ready. (DOCX)

S2 Table RD estimates on other outcomes. (DOCX)

S3 Table Narrowing the window width. (DOCX)

S4 Table Share of cash benefit recipients weeks 15 to 32. (DOCX)

S5 Table RD estimates using alternative response time weeks. (DOCX)

S6 Table Descriptive sample statistics. (DOCX)

S7 Table Gender estimates. (DOCX)
