# Supplementary material for: Incentive effects of cash benefit among low-skilled young adults: Applying a regression discontinuity design
Source: PLoS One. 2020 Nov 2;15(11):e0241279. doi: 10.1371/journal.pone.0241279 (PMC7605669; doi:10.1371/journal.pone.0241279)
Supplement: S1 Table — (DOCX) [file pone.0241279.s001.docx]

The DREAM register allows making a distinction between rates of passive and active cash benefit recipients. Being registered as an active cash benefit recipient indicates that the young adult participates in active measures such as counselling, ordinary education, subsidised work or job training. This distinction captures a potential effect among the activity-ready young adults at age 30 in moving from active to passive benefit recipient.

**S1 Table. RD estimates on passive and active activity-ready.** RD estimates of the effect at age 30 when distinguishing between passive and active cash benefit. Young adults categorized as activity-ready.

|  | First degree polynomial | Second degree polynomial | Third degree polynomial | Local polynomial (using Rdrobust) |
| --- | --- | --- | --- | --- |
| **Activity-ready – no response time** |  |  |  |  |
| Cash benefit as passive recipient | 0.052*** (0.007) | 0.034*** (0.010) | 0.034**  (0.013) | 0.044*** (0.011) |
| Cash benefit as active recipient | -0.059*** (0.006) | -0.016  (0.009) | -0.028*  (0.012) | -0.009  (0.014) |
| **Response time** |  |  |  |  |
| Cash benefit as passive recipient | 0.053*** (0.007) | 0.047*** (0.010) | 0.032*  (0.013) | 0.048*** (0.011) |
| Cash benefit as active recipient | -0.073*** (0.006) | -0.042*** (0.009) | -0.017  (0.012) | -0.013  (0.016) |

Standard errors in parentheses, *** p<0.001, ** p<0.01, * p<0.05

When including the distinction between passive and active cash benefit rates we find an increase in the share of passive benefit recipients at age 30 reflecting a jump of 3.4 to 5.2 percentage points. Including response time the effect remains the same in size and significance. This result indicates that some young adults remain in the system when they turn 30, but change status from active (= participating in labour market interventions) to passive benefit recipient.
