# Supplementary material for: Incentive effects of cash benefit among low-skilled young adults: Applying a regression discontinuity design
Source: PLoS One. 2020 Nov 2;15(11):e0241279. doi: 10.1371/journal.pone.0241279 (PMC7605669; doi:10.1371/journal.pone.0241279)
Supplement: S2 Table — (DOCX) [file pone.0241279.s002.docx]

The appendix includes effect estimates on outcomes of employment and self-support (by other means, i.e. neither receiving income transfers, in education nor in employment). For young adults with low paid jobs close to age 30 the increase in benefit might represent a disincentive to continue working. For others the prospect of taking up education might seem unmanageable, implying they look for alternative means of support. In both cases, we would expect to see a drop in levels of employment and/or self-support at age 30. In the following we only present results by visitation categories.

**S2 Table. RD estimates on other outcomes.** RD estimates of the effect of increased benefits at age 30 on self-support and employment. Young adults with low educational qualifications, by visitation category.

| **No visitation category** | First degree polynomial | Second degree polynomial | Third degree polynomial | Local polynomial (using Rdrobust) |
| --- | --- | --- | --- | --- |
| Self-support | -0.007** (0.002) | -0.001 (0.003) | -0.003 (0.004) | -0.001 (0.004) |
| Work | -0.001 (0.002) | 0.001 (0.003) | 0.003 (0.004) | -0.001 (0.004) |
| **Response time** |  |  |  |  |
| Self-support | -0.005* (0.002) | -0.007* (0.003) | -0.004 (0.005) | -0.005 (0.005) |
| Work | -0.006** (0.002) | 0.002 (0.003) | 0.001 (0.004) | 0.001 (0.005) |
| Number of included weekly observations = 755.990 (no response time); 710.363 (response time)  Number of individuals = 4.059 | | | | |
| **Education-ready** |  |  |  |  |
| Self-support | -0.013** (0.005) | -0.036 (0.007) | -0.033*** (0.009) | -0.023* (0.011) |
| Work | -0.035*** (0.006) | -0.033*** (0.009) | -0.016 (0.012) | -0.012 (0.013) |
| **Response time** |  |  |  |  |
| Self-support | -0.016*** (0.005) | -0.001 (0.007) | -0.022* (0.009) | -0.031** (0.011) |
| Work | -0.019** (0.006) | -0.025** (0.009) | -0.035** (0.012) | -0.008 (0.015) |
| Number of included weekly observations = 74.395 (no response time); 69.656 (response time)  Number of individuals = 403 | | | | |
| **Activity-ready** |  |  |  |  |
| Self-support | -0.001 (0.003) | -0.007 (0.005) | -0.013 (0.007) | -0.013 (0.007) |
| Work | 0.008* (0.003) | -0.000 (0.005) | 0.002 (0.006) | -0.002 (0.006) |
| **Response time** |  |  |  |  |
| Self-support | 0.003 (0.003) | -0.001 (0.005) | -0.006 (0.007) | -0.006 (0.007) |
| Work | 0.004 (0.003) | -0.015** (0.005) | -0.017** (0.006) | -0.014* (0.007) |
| Number of included weekly observations = 87.947 (no response time); 82.272 (response time)  Number of individuals = 474 | | | | |

*Standard errors in parentheses, *** p<0.001, ** p<0.01, * p<0.05*

The table indicates that some of the increase in cash benefit, not explained by the drop in education rates, might be associated with a small decrease in the share of self-supported young adults. This result indicates a group for whom entering education and employment has not been possible. Instead the policy change left them self-supported for a shorter period until returning to cash benefit at age 30. This is exclusively found among education-ready young adult. With and without response time this outcome shows significant negative estimates for all specifications besides the second degree polynomial in the range between -1.6 and -3.3 percent points. Further, the table indicates that employment rates drop between 1.9 to 3.5 percentage points among education-ready and between 1.4 to 1.7 percentage points for activity-ready (including response time).
