# Supplementary material for: Incentive effects of cash benefit among low-skilled young adults: Applying a regression discontinuity design
Source: PLoS One. 2020 Nov 2;15(11):e0241279. doi: 10.1371/journal.pone.0241279 (PMC7605669; doi:10.1371/journal.pone.0241279)
Supplement: S3 Table — (DOCX) [file pone.0241279.s003.docx]

In this section we narrow the window width to explore the robustness when including fewer observations to fit the model. In the following we present estimates using a window width of 50 weekly observations on each side of the age-threshold. we only include estimates when allowing for 20 weeks of response time.

**S3 Table. Narrowing the window width.** Regression discontinuity estimates of the effect of increased benefits at age 30. Young adults with low educational qualifications. Response time = 20 weeks and window width = 50 weeks

|  | **First degree polynomial** | **Second degree polynomial** | **Third degree polynomial** | **Local polynomial (using Rdrobust)** |
| --- | --- | --- | --- | --- |
| Cash benefit | 0.008*** (0.002) | 0.010*** (0.003) | 0.010** (0.003) | 0.007* (0.003) |
| Education | -0.010*** (0.001) | -0.008*** (0.002) | -0.007* (0.003) | -0.007** (0.003) |
| Self-support | -0.006 (0.003) | -0.008* (0.004) | -0.011 (0.006) | -0.006 (0.006) |
| Work | -0.005 (0.003) | -0.003 (0.004) | 0.001 (0.005) | 0.000 (0.006) |
| Number of included weekly observations = 478.185 | | | | |
| **Male** |  |  |  |  |
| Cash benefit | 0.016*** (0.003) | 0.017*** (0.004) | 0.013** (0.005) | 0.011* (0.005) |
| Education | -0.015*** (0.002) | -0.011*** (0.003) | -0.007 (0.004) | -0.009** (0.003) |
| Self-support | -0.005 (0.004) | -0.006 (0.006) | -0.006 (0.007) | -0.001 (0.007) |
| Work | -0.010* (0.004) | -0.009 (0.006) | -0.005 (0.008) | -0.005 (0.007) |
| Number of included weekly observations = 245.856 | | | | |
| **Female** |  |  |  |  |
| Cash benefit | 0.000 (0.002) | 0.004 (0.004) | 0.006 (0.005) | 0.005 (0.004) |
| Education | -0.005* (0.002) | -0.006 (0.004) | -0.007 (0.005) | -0.006 (0.004) |
| Self-support | -0.007 (0.004) | -0.013 (0.007) | -0.018* (0.009) | -0.013 (0.009) |
| Work | 0.001 (0.004) | 0.003 (0.006) | 0.008 (0.007) | 0.006 (0.008) |
| Number of included weekly observations = 202.146 | | | | |
| **No category** |  |  |  |  |
| Cash benefit | 0.001*** (0.000) | 0.001* (0.000) | 0.000 (0.000) | 0.000 (0.000) |
| Education | -0.001 (0.001) | 0.000 (0.002) | 0.001 (0.003) | -0.001 (0.002) |
| Self-support | -0.006 (0.003) | -0.008 (0.005) | -0.007 (0.006) | -0.003 (0.006) |
| Work | -0.002 (0.003) | 0.001 (0.005) | 0.002 (0.006) | 0.002 (0.006) |
| Number of included weekly observations = 393.269 | | | | |
| **Activity-ready** |  |  |  |  |
| Cash benefit | -0.001 (0.009) | 0.012 (0.013) | 0.010 (0.017) | 0.015 (0.018) |
| Passive | 0.042*** (0.009) | 0.046*** (0.014) | 0.026 (0.019) | 0.021 (0.021) |
| Active | -0.043*** (0.008) | -0.034** (0.012) | -0.016 (0.016) | -0.011 (0.018) |
| Education | -0.039*** (0.005) | -0.036*** (0.007) | -0.029** (0.010) | -0.023* (0.011) |
| Self-support | -0.000 (0.005) | -0.005 (0.007) | -0.009 (0.009) | -0.010 (0.010) |
| Work | -0.010* (0.004) | -0.018** (0.007) | -0.020* (0.009) | -0.008 (0.011) |
| Number of included weekly observations = 46.002 | | | | |
| **Education-ready** |  |  |  |  |
| Cash benefit | 0.085*** (0.010) | 0.108*** (0.014) | 0.096*** (0.019) | 0.059** (0.023) |
| Education | -0.065*** (0.008) | -0.063*** (0.012) | -0.058*** (0.015) | -0.044** (0.016) |
| Self-support | -0.009 (0.006) | -0.025* (0.010) | -0.046*** (0.013) | -0.031 (0.016) |
| Work | -0.030*** (0.009) | -0.027* (0.013) | 0.015 (0.017) | -0.007 (0.016) |
| Number of included weekly observations = 38.914 | | | | |

Standard errors in parentheses, *** p<0.001, ** p<0.01, * p<0.05

The table demonstrates how the findings largely remain intact: cash benefit and education rates express a similar relation, the relation is significant for males only and again, education-ready young adults are the main drivers of the effect. The relation between passive and active benefits found above is confirmed as well when narrowing the window width.
