# Supplementary material for: Incentive effects of cash benefit among low-skilled young adults: Applying a regression discontinuity design
Source: PLoS One. 2020 Nov 2;15(11):e0241279. doi: 10.1371/journal.pone.0241279 (PMC7605669; doi:10.1371/journal.pone.0241279)
Supplement: S4 Table — (DOCX) [file pone.0241279.s004.docx]

**S4 Table. Share of cash benefit recipients weeks 15 to 32.**

The table describe the development in the weekly share of cash benefit recipients among young adults with low educational qualifications

| **Week** | **Share of cash benefit recipient** |
| --- | --- |
| 15 | 0.0967 (9.67 percent) |
| 16 | 0.0969 (9.69 percent) 1. PEAK |
| 17 | 0.0963 (9.63 percent) |
| 18 | 0.0967 (9.67 percent) |
| 19 | 0.0965 (9.65 percent) |
| 20 | 0.0961 (9.61 percent) |
| 21 | 0.0969 (9.69 percent) 2. PEAK |
| 22 | 0.0967 (9.67 percent) |
| 23 | 0.0967 (9.67 percent) |
| 24 | 0.0969 (9.69 percent) 3. PEAK |
| 25 | 0.0963 (9.63 percent) |
| 26 | 0.0963 (9.63 percent) |
| 27 | 0.0959 (9.59 percent) |
| 28 | 0.0967 (9.67 percent) |
| 29 | 0.0963 (9.63 percent) |
| 30 | 0.0955 (9.55 percent) |
| 31 | 0.0963 (9.63 percent) |
| 32 | 0.0951 (9.51 percent) |
