# Supplementary material for: Incentive effects of cash benefit among low-skilled young adults: Applying a regression discontinuity design
Source: PLoS One. 2020 Nov 2;15(11):e0241279. doi: 10.1371/journal.pone.0241279 (PMC7605669; doi:10.1371/journal.pone.0241279)
Supplement: S5 Table — (DOCX) [file pone.0241279.s005.docx]

**S5 Table. RD estimates using alternative response time weeks.**

The tables presented in this appendix are similar to Table 3 (RD estimates for young adults with low educational qualifications.), but use alternative response time weeks. The appendix presents estimates when using response time set to week 16, 24 and 28.

**Table 3a – response time set to 16 weeks**

|  | | **First degree polynomial** | **Second degree polynomial** | **Third degree polynomial** | **Local polynomial (using Rdrobust)** |
| --- | --- | --- | --- | --- | --- |
| **No response time** | Cash benefit | 0.005***  (0.001) | 0.006**  (0.002) | 0.005*  (0.002) | 0.004*  (0.002) |
|  | Education | -0.007***  (0.001) | -0.004**  (0.001) | -0.000  (0.002) | -0.001  (0.002) |
| **Response time (16 weeks)** | Cash benefit | 0.006***  (0.001) | 0.008***  (0.002) | 0.011***  (0.002) | 0.009***  (0.002) |
|  | Education | -0.010*** (0.001) | -0.010*** (0.001) | -0.008*** (0.002) | -0.007**  (0.002) |

**Table 3b – response time set to 24 weeks**

|  | | **First degree polynomial** | **Second degree polynomial** | **Third degree polynomial** | **Local polynomial (using Rdrobust)** |
| --- | --- | --- | --- | --- | --- |
| **No response time** | Cash benefit | 0.005***  (0.001) | 0.006**  (0.002) | 0.005*  (0.002) | 0.004*  (0.002) |
|  | Education | -0.007***  (0.001) | -0.004**  (0.001) | -0.000  (0.002) | -0.001  (0.002) |
| **Response time (24 weeks)** | Cash benefit | 0.005***  (0.001) | 0.015***  (0.003) | 0.010***  (0.002) | 0.008***  (0.002) |
|  | Education | -0.010*** (0.001) | -0.015*** (0.002) | -0.009*** (0.002) | -0.007**  (0.002) |

**Table 3c – response time set to 28 weeks**

|  | | **First degree polynomial** | **Second degree polynomial** | **Third degree polynomial** | **Local polynomial (using Rdrobust)** |
| --- | --- | --- | --- | --- | --- |
| **No response time** | Cash benefit | 0.005***  (0.001) | 0.006**  (0.002) | 0.005*  (0.002) | 0.004*  (0.002) |
|  | Education | -0.007***  (0.001) | -0.004**  (0.001) | -0.000  (0.002) | -0.001  (0.002) |
| **Response time (28 weeks)** | Cash benefit | 0.004***  (0.001) | 0.006**  (0.002) | 0.010***  (0.002) | 0.008***  (0.002) |
|  | Education | -0.010*** (0.001) | -0.011*** (0.001) | -0.009*** (0.002) | -0.007**  (0.002) |
