# Supplementary material for: Incentive effects of cash benefit among low-skilled young adults: Applying a regression discontinuity design
Source: PLoS One. 2020 Nov 2;15(11):e0241279. doi: 10.1371/journal.pone.0241279 (PMC7605669; doi:10.1371/journal.pone.0241279)
Supplement: S6 Table — (DOCX) [file pone.0241279.s006.docx]

**S6 Table. Descriptive sample statistics.** The Table includes central statistics for the full population of young adults turning 30 during 2014 or 2015 (13.413 young adults) and for those who have low educational qualifications (4.936 young adults).

|  | **Full sample of young adults turning 30 in 1984 and 1985** | **Sample of young adults with low educational qualifications** |
| --- | --- | --- |
|  | Percent *(number)* | Percent *(number)* |
| **Birth year** |  |  |
| 1984 | 49.6 | 49.9 |
| 1985 | 50.4 | 50.1 |
| **Gender** |  |  |
| Male | 50.2 | 51.4 |
| Female | 47.5 | 42.3 |
| Missing gender | 2.3 | 6.3 |
| **Family status**  **(measured at age 29)** |  |  |
| Cohabiting or married | 50.7 | 33.9 |
| Single | 37.1 | 34.9 |
| Unknown family status | 12.3 | 31.2 |
| **Parent at age 29** | 22.2 | 15.3 |
| **Ethnicity** |  |  |
| Danish | 72.0 | 42.7 |
| 1. generation immigrant | 25.7 | 54.0 |
| 2. generation immigrant | 2.3 | 3.2 |
| **Highest attained education level**  **(measured at age 29)** |  |  |
| Primary and lower secondary | 30.6 | 81.3 |
| Upper secondary | 5.8 | 18.7 |
| Vocational | 25.4 | 0 |
| Short higher education | 4.9 | 0 |
| Bachelor | 17.4 | 0 |
| Master and doctoral | 15.8 | 0 |
| **Visitation category**  **(measured after 2014)** |  |  |
| No visitation category |  | 82.2 (4.059) |
| Education-ready |  | 8.2 (403) |
| Activity-ready |  | 9.6 (474) |
| **Total** | **13.413** | **4.936** |

Source: Own calculations based on registers from Statistics Denmark
