# Supplementary material for: Incentive effects of cash benefit among low-skilled young adults: Applying a regression discontinuity design
Source: PLoS One. 2020 Nov 2;15(11):e0241279. doi: 10.1371/journal.pone.0241279 (PMC7605669; doi:10.1371/journal.pone.0241279)
Supplement: S7 Table — (DOCX) [file pone.0241279.s007.docx]

**S7 Table. Gender estimates.** RD estimates of the effect of increased benefits at age 30 on different outcomes. Young adults with low educational qualifications, by gender

| **Men – no response time** | First degree polynomial | Second degree polynomial | Third degree polynomial | Local polynomial (using Rdrobust) |
| --- | --- | --- | --- | --- |
| Cash benefit | 0.011***  (0.002) | 0.011***  (0.003) | 0.008*  (0.004) | 0.006  (0.004) |
| Education | -0.007*** (0.001) | -0.005**  (0.002) | 0.000  (0.003) | -0.000  (0.003) |
| **Response time** |  |  |  |  |
| Cash benefit | 0.012***  (0.002) | 0.014***  (0.003) | 0.019***  (0.004) | 0.015***  (0.003) |
| Education | -0.013*** (0.001) | -0.015*** (0.002) | -0.014*** (0.003) | -0.009**  (0.003) |
| Number of included weekly observations = 472.015 (no response time); = 443.464 (response time) | | | | |
| **Women – no response time** |  |  |  |  |
| Cash benefit | -0.001  (0.002) | 0.001  (0.003) | 0.001  (0.004) | 0.002  (0.004) |
| Education | -0.007*** (0.002) | -0.004  (0.002) | -0.001  (0.003) | -0.002  (0.003) |
| **Response time** |  |  |  |  |
| Cash benefit | -0.001  (0.002) | 0.000  (0.003) | 0.004  (0.004) | 0.005  (0.004) |
| Education | -0.009*** (0.002) | -0.007**  (0.002) | -0.004  (0.003) | -0.005  (0.003) |
| Number of included weekly observations = 364.339 (response time); = 338.262 (no response time) | | | | |

Standard errors in parentheses, *** p<0.001, ** p<0.01, * p<0.05

Introducing gender reveals that the global effect on cash benefit is present for males only and when allowing for time to adapt to the policy change the increase seems closely linked to decreasing educational activity. Further, the effects among males are more extensive with estimates of cash benefit rates reaching an increase of up to almost 2 percent points and a corresponding estimated decrease in education level of up to 1.5 percent points. For women the lower polynomial specifications indicate significant effects on decreased educational activity. Despite not being significant both effect size and direction is further confirmed by the most flexible specifications.
